# 

**Published:** 1895-06

**Authors:** 


					CONTENTS.
Alcoholic Disease of the Heart.
By
l heodore Fisher, M.D. Lond.
The Paralysis of Osteo-Arthritis.
By
henry Waldo, M.D. Aberd.,
M.R.C.P. Lond
90
Buccal Glandular Tumours.
ByJames
Swain, M.D., M.S. Lond., F.R.C.S.
Eng
94
Sarcomatous Tumour of the Shoulder-
Centre in the Cortex of the Right
nemispnere.
By J. E. Shaw, M.B.
Ed., and J. Paul Bush, M.R.C.S. Eng.
99
Four Cases of Blood - Poisoning in
By F. H. Edgeworth.
?ts.A., M.B. Cantab., B.Sc. Lond.
Pneumococci in the Urine.
By tx.
Munro Smith, M.K.C.b. ??ng.,
L.R.C.P. Lond. ...
lis
Progress of the Medical Sciences:
Medicine.
By George Parker, M.D.
Surgery.
By Charles A. Morton
129
Obstetrics.
By L. M. Griffiths
Reviews of Books
141
Notes on Preparations for the Sick
149
Meetings of Societies
151
The Library of the Bristol Medico-
Chirurgical Society 13d
Local Medical Notes
158
Scraps
159

				

